# The palisade layer of the poxvirus core is composed of flexible A10 trimers

**DOI:** 10.1038/s41594-024-01218-5

**Published:** 2024-02-05

**Authors:** Jiasui Liu, Simon Corroyer-Dulmont, Vojtěch Pražák, Iskander Khusainov, Karola Bahrami, Sonja Welsch, Daven Vasishtan, Agnieszka Obarska-Kosińska, Sigurdur R. Thorkelsson, Kay Grünewald, Emmanuelle R. J. Quemin, Beata Turoňová, Jacomina Krijnse Locker

**Affiliations:** 1 Department of Molecular Sociology, Max Planck Institute of Biophysics, Frankfurt am Main, Germany; 2 Centre for Structural Systems Biology, Leibniz Institute of Virology, Hamburg, Germany; 3 University of Hamburg, Hamburg, Germany; 4 Department of Biochemistry, University of Oxford, Oxford, UK; 5 Electron Microscopy of Pathogens, Paul Ehrlich Institute, Langen, Germany; 6 Max Planck Institute of Biophysics, Central Electron Microscopy Facility, Frankfurt am Main, Germany; 7 Division of Structural Biology, Wellcome Centre for Human Genetics, University of Oxford, Oxford, UK; 8 Department of Virology, Institute for Integrative Biology of the Cell (I2BC), CNRS UMR9198, Université Paris-Saclay, CEA, Gif-sur-Yvette, France; 9 Justus Liebig University of Giessen, Giessen, Germany; 10 Present Address: University Clinic Frankfurt, Frankfurt am Main, Germany; 11 Present Address: MRC Laboratory of Molecular Biology, Cambridge, UK

**Keywords:** Cryoelectron tomography, Virology, Cellular imaging

## Abstract

Due to its asymmetric shape, size and compactness, the structure of the infectious mature virus (MV) of vaccinia virus (VACV), the best-studied poxvirus, remains poorly understood. Instead, subviral particles, in particular membrane-free viral cores, have been studied with cryo-electron microscopy. Here, we compared viral cores obtained by detergent stripping of MVs with cores in the cellular cytoplasm, early in infection. We focused on the prominent palisade layer on the core surface, combining cryo-electron tomography, subtomogram averaging and AlphaFold2 structure prediction. We showed that the palisade is composed of densely packed trimers of the major core protein A10. Trimers display a random order and their classification indicates structural flexibility. A10 on cytoplasmic cores is organized in a similar manner, indicating that the structures obtained in vitro are physiologically relevant. We discuss our results in the context of the VACV replicative cycle, and the assembly and disassembly of the infectious MV.

## Abstract (web summary)

The authors employ cryo-ET to study the structure of the palisade layer of the mature vaccinia virus core in isolation, as well as inside infected cells, revealing that it is composed of A10 protein trimers.

## Main

The recent emergence of monkeypox virus infections in Europe and North America has rekindled an interest in poxviruses. The prototype of the poxviruses, vaccinia virus (VACV), was used previously as a live vaccine to eradicate smallpox and has been studied biochemically, genetically and morphologically (reviewed in ref. ^1^). However, despite extensive investigation, key questions remain about the biogenesis and structure of the mature virus (MV) (previously also referred to as IMV, intracellular mature virus)^2^.

The infectious MV is a quasi-brick-shaped particle measuring roughly 250 nm × 350 nm × 200 nm. It is composed of an oval core enclosing the viral genome as well as the machinery required for transcription early in infection. This core is surrounded by a lipid bilayer acquired during assembly; two so-called lateral bodies are located on the elongated sides of the core brick, underneath the viral membrane^3^. The MV is composed of up to 200 proteins^4^. Treatment of MVs with a nonionic detergent and a reducing agent results in a membrane-free stable core^5^, which has been widely used to study VACV proteins associated with the membrane and the core. The membrane-free core is known to be composed of at least five abundant proteins: the gene products of A3, A4, A10, L4 and F17 (ref. ^2^). With the exception of F17, these proteins are also associated with the incoming cytoplasmic core, early in infection^6^.

Although the MV has been studied extensively by electron microscopy (EM), including cryo-EM, its precise structure and molecular composition remain elusive. Structural analyses are hampered in particular by the compactness of the virion and its asymmetric shape. Thus, subviral structures have also been studied, in particular the membrane-free and detergent-stripped core, by negative-staining EM^7–9^ and by cryo-EM^10^. Such cores display a dense, prominent layer on their surface, the so-called palisade. Early cryo-EM data suggested that the palisade units are hollow tubes, roughly 20 nm in length and 10 nm in diameter, and appear as tightly packed tubes on the core surface forming a hexagonal lattice with a 9.7 nm spacing that was partially disordered in some regions^10^. On the basis of analysis of the intact MV by cryo-electron tomography (cryo-ET), it was proposed that the palisade units on the core surface are 8 nm in length and 5 nm in diameter and arranged as hexagonal lattices^3^. A more recent publication studying VACV assembly by cryo-ET in the periphery of infected cells also proposed an ordered arrangement of the palisade on fully assembled virions^11^. The model proposed in this study displays the palisade as trimeric pillars with projecting lobes that mediate interpillar contacts.

Although the major proteins associated with membrane-free cores are known, it is unclear which of these proteins make up its prominent features, such as the core wall and the characteristic palisade^2^. Immunolabeling experiments suggested that the palisade is composed of the gene product of A3 (ref. ^8^). In contrast, using controlled degradation of the membrane-free core, the gene product of A4 was proposed as the palisade protein^9^.

Here we apply cryo-ET and subtomogram averaging (STA) to study the structure and molecular composition of the palisade both in vitro and in situ. From the in vitro data we obtain a structure of the palisade unit in two conformations, with resolutions of 8.5 Å and 7.7 Å. Both structures are also seen on the palisade in situ, on the surface of the incoming core early in infection, arguing that they are physiologically relevant. We apply AlphaFold2 (ref. ^12^) to predict the three-dimensional (3D) structure of the five major core proteins, A3, A4, A10, L4 and F17; by fitting the predicted structure of the gene product of A10 into the obtained map, we show that the palisade units are made up of trimers of this core protein. The relevance of these data, including additional features we observe in our cryo-tomograms, is discussed with respect to the biology of poxvirus infection.

## Results

### VACV core sample preparation for cryo-ET

**Extended Data Fig. 1:**
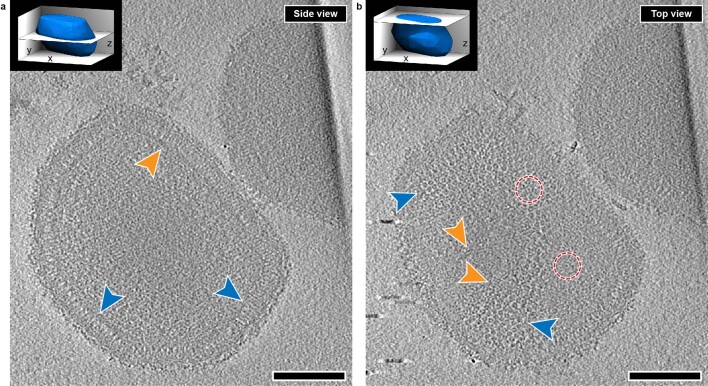
Tomogram slices of purified mature virions (MV) of VACV. (**a**) Slice of a tomogram (shown in Movie S1) showing the cross-section of a MV. Blue arrowheads show side views of the palisade layer. The orange arrowhead points to the side view of the core wall underneath the palisade layer. (**b**) Slice of the same tomogram close to the top of the MV, showing top views of the palisade (blue arrowheads), the core wall (orange arrowheads) and the ring-like structures (red circles). Scale bars, 100 nm. Insets indicate the rough positions of the respective tomogram slices w.r.t the MV.

For structural analysis of the VACV core by cryo-ET, sample preparation was optimized to obtain intact cores in vitro (Methods). Low NP-40 and dithiothreitol (DTT) concentrations, diluted in 50 mM Tris–Cl pH 8.5, appeared optimal, resulting in cores that are structurally intact and remain associated with lateral bodies. Inspection of vitrified samples and selection of suitable areas for cryo-ET resulted in 34 tilt series containing 50 cores that were used for further analysis. Extended Data Fig. 1 and Supplementary Video 1 display a typical MV from purified VACV core preparation, exemplifying the complexity of its structure.

### The VACV in vitro cores display three layers

**Fig. 1:**
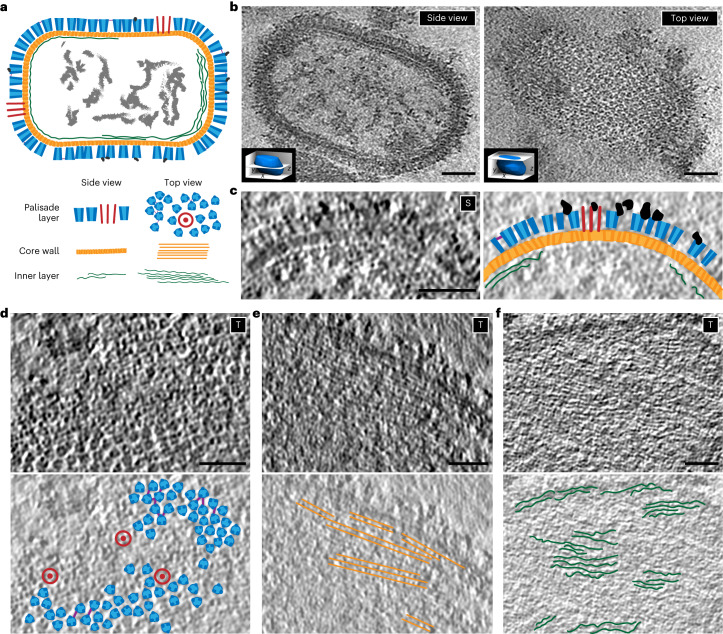
Organization of VACV core prepared in vitro by cryo-ET. **a**, Cartoon representation of the in vitro VACV core based on visual analysis of tomograms, depicting a slice through the center of the core (top) and details of the three layers from the side and top view (bottom). The red structures, displayed as circles in the top view, represent the ring-like structures. **b**, Digital slices of a representative tomogram with side (left) and top (right) views of the in vitro core. The top view reveals a palisade made of tube-like structures, the stakes, that have an irregular arrangement. The side view of the core reveals three layers, that is, the palisade, the core wall and the innermost layer, and sparse densities in the inner part of the core. Insets indicate the rough positions of the respective tomogram slices with respect to the core. **c–f**, Details of the three different layers surrounding the inner part of the core from the side (S) and the top (T) views. **c**, Detail of the organization from the side view (left) and its corresponding annotation (right). The dark blobs depict the strong density occasionally observed on top of the palisade stakes. **d**, Detail of the palisade layer at the surface of the core from the top view (top) and its corresponding annotation (bottom): the palisade stakes in blue with connections between them in violet and the ring-like structures in red. **e**, Detail of the core wall layer from the top view (top) and its corresponding annotation showing the stripe-like pattern in orange (bottom). **f**, Detail of the innermost layer of the core wall from the top view (top) and its corresponding annotation in green (bottom). Scale bars, 30 nm.

Reconstructed tomograms of the VACV cores, obtained after detergent stripping of intact virus, confirm the overall organization observed previously (Fig. 1a,b and Supplementary Video 2)^10^. The core acquires a brick-like shape with average dimensions of 200 nm × 320 nm × 200 nm. The inside of the core is sparsely filled with electron densities, possibly representing the viral genome in complex with DNA-binding proteins or the transcription machinery packaged during assembly^13^. It is enclosed by at least three layers (Fig. 1b); the most prominent is the outer palisade layer composed of protrusions with a tube-like shape of ~11 nm in height and a diameter of 8 nm (Fig. 1b). Being subunits of the palisade, we will refer to these tubular structures as stakes throughout this study. As shown in more detail below, the stakes do not form a regular lattice (Fig. 1b,d) in contrast to what has been described previously^10,11,14^. At their top, the stakes are often connected to their neighbors with thin threads (Fig. 1d). A ring structure previously reported in ref. ^11^ was found integrated into the palisade (Fig. 1c,d). Below the palisade is the middle layer, the inner core wall with a thickness of 4–5 nm (Fig. 1c), which appears as stripes when viewed from the top (Fig. 1e). Interestingly, the arrangements of the stakes in the palisade layer and the stripes in the inner core wall layer do not seem to be aligned (Supplementary Video 2). The innermost layer is roughly 3 nm below the inner core wall and has a thickness of less than 2 nm. The content of this layer appears irregular within each core: it can contain one or several layers (Fig. 1c,f) and is sometimes even absent. Strong densities are occasionally found to be connected to the top of the stakes (Fig. 1c). Consistent with previous data and the gentle sample preparation procedure we used, these in vitro cores also contain two large blob-like structures on both sides: the lateral bodies. In our tomograms the lateral bodies appear without higher-order organization, although connections linking them to the stakes of the palisade layer could be observed (Fig. 1b and Supplementary Video 2).

### AlphaFold2 predictions of major core proteins

**Fig. 2:**
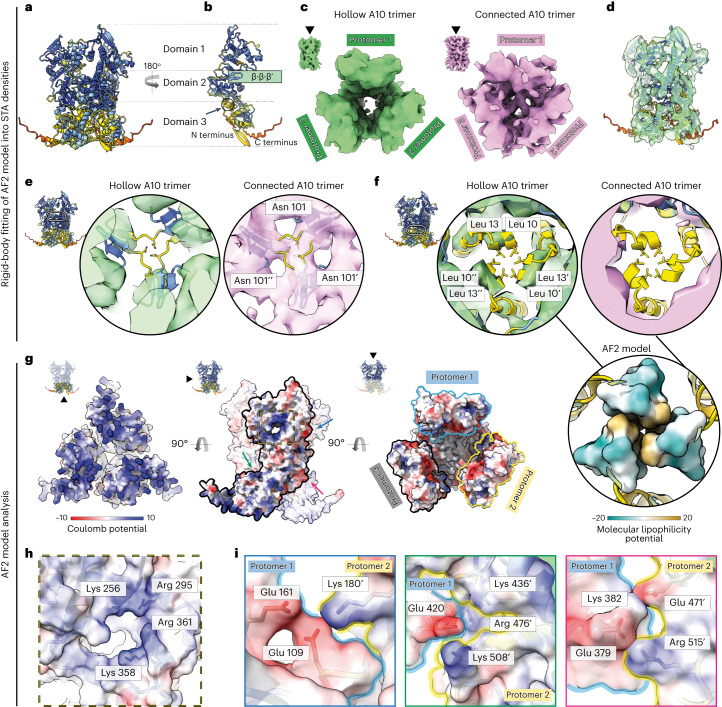
Classification and structural analysis of the A10 protein. **a**, AF2 prediction of the A10 trimer colored by per-residue confidence score (pLDDT) between 0 (red) and 100 (dark blue). **b**, Interface view of an individual protomer from the AF2-predicted A10 trimer. The β-strands that correspond to the linker region of a connection trimer are highlighted with the green rectangle and labeled as β·β·β′, referring to a putative β-sheet composed of two β-strands from one protomer and one β-strand from another protomer. The black arrow and the circle indicate the location of an α-helix that forms putative interprotomer hydrophobic interactions at the domain 3 region. **c**, STA of the two distinct A10 trimer classes, hollow (green) and connected (pink). **d**, AF2-predicted model of A10 trimer, rigid body-fitted into the STA map of a hollow trimer. **e**, STA maps of the hollow (left) and connected (right) A10 trimers with the rigid body-fitted AF2-predicted model, sliced at the domain 2 region and showing the density at the region of putative β-sheet formation and interaction between Asn101 residues of each protomer. **f**, STA maps of the hollow (left) and connected (right) A10 trimers with the rigid body-fitted AF2-predicted model, sliced at the domain 3 region and showing α-helices forming putative hydrophobic interactions. The bottom panel represents a hydrophobic interface formed by the N-terminal residues Leu10 and Leu13 of each protomer. The coloring is from dark cyan (most hydrophilic, −20 e^–d^,) to dark gold (most hydrophobic, 20 e^–d^,). The values correspond to the molecular lipophilicity potential calculated by the Fauchére method. **g**, Electrostatic potential of the AF2-predicted model of the A10 trimer (negative potential in red, positive in blue). The three subpanels correspond to bottom view (left), side view (middle) and top view (right) of an A10 trimer. **h**, The positively charged cavity on the solvent side of the A10 protomer. Coloring as in **g**. **i**, Interprotomer electrostatic interactions observed on the AF2-predicted model of the A10 trimer. Coloring of models and surfaces as in **g**. Colors of contours of each subpanel correspond to the arrow colors in **g**.

**Extended Data Fig. 2:**
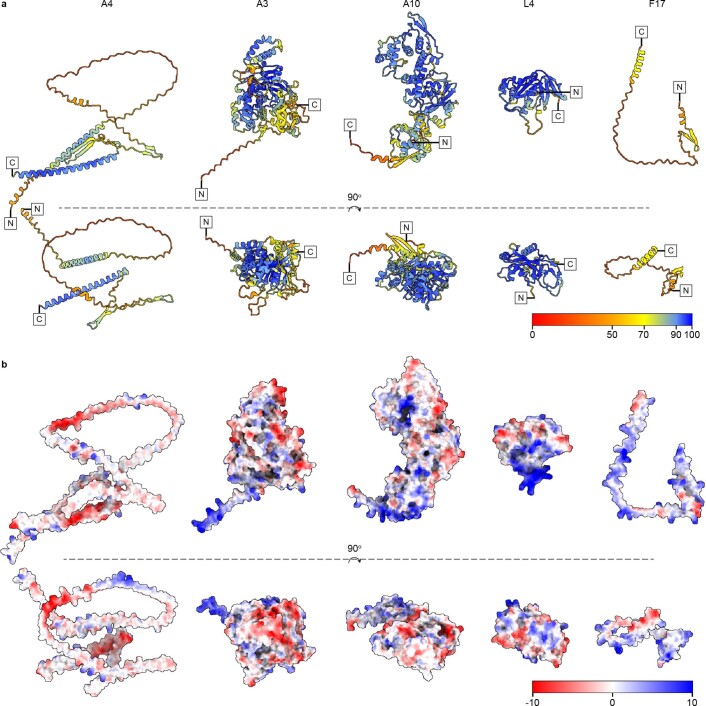
Structures of VACV protein monomers predicted with AlphaFold2 software. The best prediction for each of the proteins A4, A3, A10, L4 and F17 is depicted at two different views. (**a**) Structures are colored according to pLDDT confidence measure between 0 (red) and 100 (blue). High accuracy is assumed with pLDDT > 90 and the backbone is expected to be modeled well with pLDDT between 70 and 90. Any region with pLDDT below 70 has low confidence and should be interpreted with caution. Regions with pLDDT below 50 should not be interpreted. Locations of C and N-termini are indicated for all predicted models. (**b**) Electrostatic surface potentials of the structure predictions depicted in (**a**) are colored red and blue for negative and positive charges, respectively. White color represents neutral residues. The visualization was done in ChimeraX.

**Extended Data Fig. 3:**
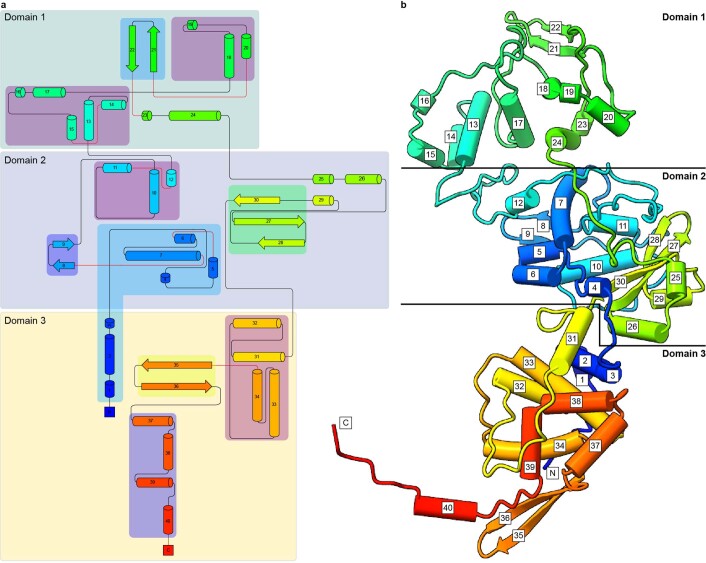
Secondary structure topology of A10 protomer. (**a**) Topology diagram of the A10 protomer fold illustrating the relative size of strands and helices, the alignment of strands in a sheet and the general positioning of sheets and helices with respect to one another. The secondary features are numbered consecutively and color-coded with a rainbow gradient from blue (N-terminus) to red (C-terminus). The non-crossing connectors are denoted in black, the crossing connectors in red. The diagram is split into three domains as indicated in Fig. 2b. The diagram was adapted from a Pro-Origami output^43^. (**b**) Cartoon diagram of A10 protomer colored from blue (N-terminus) to red (C-terminus). Strand and helix numbering is identical to that of the topology diagram in (**a**).

AlphaFold2 (AF2 (ref. ^12^)) was used to predict the 3D structure of five viral proteins known to be abundant in fractions of cores prepared in vitro^5^. These are the gene products of A3, A4, A10 and L4, and the lateral body protein F17 (ref. ^6^). The predictions were computed for various assemblies, from monomers to hexamers and different combinations of the proteins (for the full list, see Extended Data Table 1). On the basis of the confidence scores (Extended Data Table 1), the monomers of A3, A10 and L4 were found to form ordered structures, while A4 and F17 are mostly disordered (Extended Data Fig. 2a). The confidence scores for multimer predictions (Extended Data Table 1) suggest that A3 and L4 could form dimers, while A10 most likely forms a trimer (Fig. 2a). We arbitrarily divided the A10 fold into three domains (Fig. 2b and Extended Data Fig. 3), with domain 1 facing the outer region of the virus, domain 2 forming the central channel of the trimer and domain 3 facing the inner core of the virus. The major structural elements within a central channel correspond to β-strands 8 and 9 and α-helix 1 (Extended Data Fig. 3).

### The palisade consists of trimers of major core protein A10

**Extended Data Fig. 4:**
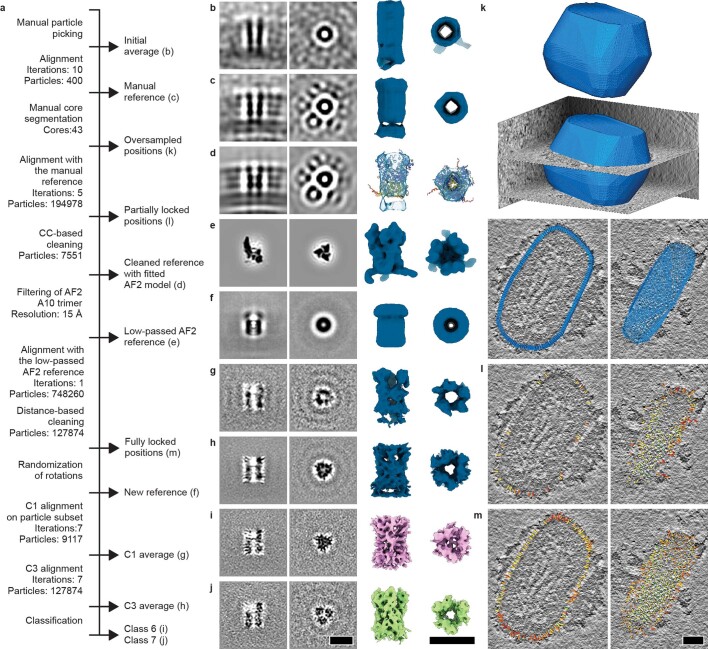
Particle picking and subtomogram averaging (STA) workflow. (**a**) Scheme of the workflow for the *in vitro* dataset. (**b**-**j**) Cross-sections of the maps obtained during processing (left) and their corresponding isosurface representations (right). The cross-sections were taken in the center of the maps for side views (leftmost column) and at domain 2 level for the top views (second to left). (**b**) Initial average obtained after manual particles picking. (**c**) The map from (**b**) after STA alignment. (**d**) The map obtained after STA of oversampled positions shown in (**k**) using the map from (**c**) as a reference, followed by constrained cross-correlation (CCC) cleaning. (**e**) Map of the AlphaFold2 (AF2) prediction of A10 trimer filtered to 15 Å. (**f**) The map obtained by randomizing the angles from the STA done on the oversampled positions using the map from (**e**) as a reference. (**g**) Map obtained by STA applying C1 symmetry on the subset of the randomized positions and with the map from (**f**) as a reference. (**h**) Map obtained by STA applying C3 symmetry on the randomized positions and using the map from (**f**) as a reference. (**i**) Map of the class 6 (connected A10 trimer). (**j**) The map of the class 7 (hollow A10 trimer). (**k**) Oversampled positions on the core surface (top panel) are depicted within a tomogram (middle panel), indicating the two cross-slices displayed in the bottom panel with the oversampled position overlay. (**l**) Partially locked positions after STA using manual reference from (**c**). The color-coding corresponds to the CCC values from the alignment; green for the high CCC values, red for the low ones. (**m**) The fully locked positions after STA on oversampled positions with the map from (**e**). The color-coding is same as in (**l**). Visualization for (**k**–**m**) was done in ChimeraX with ArtiaX module. Scale bars 10 nm for (**b**-**j**), 50 nm for (**k**–**m**).

**Extended Data Fig. 5:**
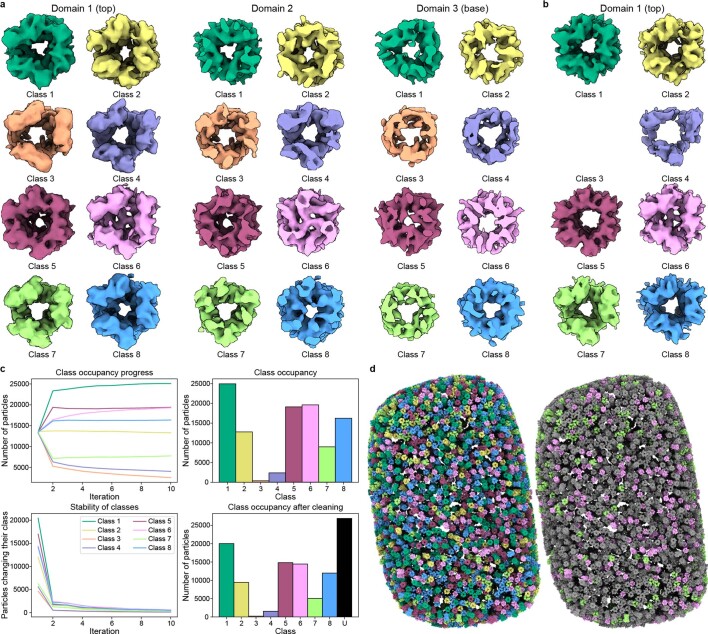
Classification of stakes from VACV *in vitro* core palisade revealing flexibility of A10 trimer. (**a**) Eight classes of *in vitro* VACV A10 trimers that were selected out of 20 classes based on visual inspection of structural differences. Three different levels are displayed: top of the trimer (Domain 1, left), middle, (Domain 2, center) and base (Domain 3, right). Differences between classes are mainly seen in the additional densities in domain 2 (class 2, 4, 6) and domain 3 (class 1, 3, 5, 8). (**b**) The final seven classes of A10 trimers, produced by 4 independent STA runs with the classes from (**a**) used as starting references. Class 3 emptied during the processing. (**c**) Graphs showing class occupancy (top right) and progress (top left) and class stability (bottom left) from a single STA run with the starting references from (**a**). Four independent and random STA runs were performed. Only particles that ended up in the same class for all 4 runs were kept (bottom right). The rest remained unassigned (U). (**d**) The final maps, plotted back into tomograms using ArtiaX plugin in ChimeraX. The color-coding corresponds to the individual classes shown in (**b**) (left panel), or to classes 6 (connected trimer) and 7 only (hollow trimer) (right panel). There is no apparent clustering or preferred position of trimers on the core palisade that would correspond to the assigned classes. Source data

**Extended Data Fig. 6:**
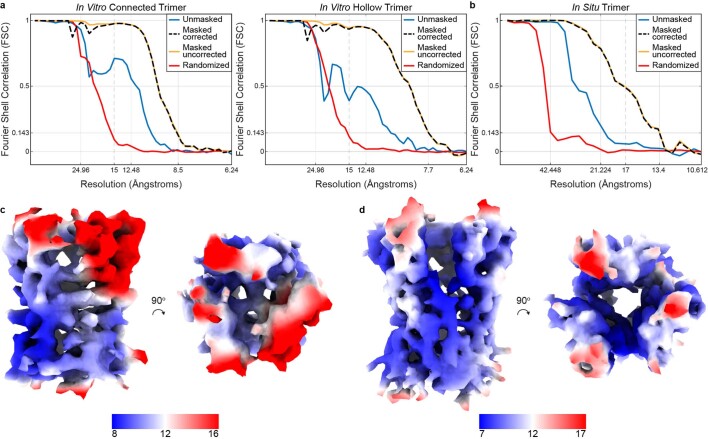
FSC curves and local resolution maps. (**a**, **b**) Four types of FSC curves are depicted for each map. The unmasked FSC was calculated without applying a mask to the half-maps. The masked uncorrected FSC was calculated on half-maps masked with a shape mask. The randomized FSC was calculated using phase-randomization^44^. The randomized FSC was used to correct the masked uncorrected FSC which resulted in the fourth FSC curve denoted as masked corrected. The difference between the masked uncorrected and unmasked curves is caused by the dense environment of the trimers. Without the mask, the signal surrounding the average contains also signal from the neighboring trimers. Since there is no regular pattern among the neighbors, the FSC decreases at lower resolution then in the presence of the mask. The addition of the phase-randomized and mask corrected FSCs shows that the mask itself is not causing strong artifactual correlations, but instead it is focusing the resolution estimation on the central trimer. All FSC curves were calculated using STOPGAP. (**a**) FSC for the connected trimer (left), which was resolved to 8.5 Å (marked in the graph) and the hollow trimer (right), resolved to 7.7 Å (marked in the graph). Both graphs show the mark for 15 Å which corresponds to the resolution of AF2 reference used for particle localization (Extended Data Fig. 4e). The resolution cut-off for phase-randomization was 30 Å. (**b**) FSC for the in situ structure. Both the map resolution (13.4 Å) and resolution of the AF2 model (17 Å) used for particle localization are marked in the graph. The resolution cut-off for phase-randomization was 59.4 Å. (**c**) STA map for the connected trimer colored by the local resolution of the best resolved regions (blue) to the worst-resolved ones (red). (**d**) STA map for the hollow trimer colored by the local resolution as in (**c**).

The initial low-resolution structure of the stakes on the core surface, obtained by STA of 400 manually picked stakes, reveals a tube-like shape with a diameter of 9 nm and a length of 11.5 nm (Extended Data Fig. 4b). From the AF2 predictions of the major core proteins, a trimer of A10 fits in shape and size to this first average (Extended Data Fig. 4d). We used the AF2-predicted A10 trimer as an initial reference to localize the stakes on all segmented cores using the oversampling approach (see Methods for more details). To limit potential template bias, the initial reference for the subsequent alignment was created using only the positions, not the orientations found during the localization (Extended Data Fig. 4f). For classification, we followed the protocol from ref. ^13^. First, we obtained 20 different de novo classes as potential candidates for further classification. On the basis of visual inspection, 8 classes stand out with substantial structural differences in the central parts of the stake (Extended Data Fig. 5a,b), while the remaining 12 classes are variations with minor differences in protomer positions and orientations, confirming the inherent flexibility of the stake structure. Following the classification protocol, the eight structurally varying classes were used as starting references for several independent classification runs (Extended Data Fig. 5b). These revealed classes with distinct features within the central part of the stake. Specifically, they were characterized by the absence or presence of densities connecting the three protomers across the inner central region (Extended Data Fig. 5b), further referred to as hollow and connected trimers, respectively (Fig. 2c). Interestingly, mapping the classes back to the tomograms shows that there is no preferential clustering or location of the different classes on the core surface (Extended Data Fig. 5d). The majority of classes forms hollow trimers and has minor differences in orientation relative to each other (Extended Data Fig. 5b). The measured overall resolution of the best hollow and connected classes reached 7.7 Å and 8.5 Å, respectively (Table 1 and Extended Data Fig. 6a).

**Table 1:** Data collection and processing parameters for all three datasets

	Intact MV	In vitro cores (EMD-17704), (EMD-17708)	In situ cores (EMD-17753)
**Data collection and processing**			
Magnification	×64,000	×81,000	×33,000
Voltage (kV)	300	300	300
Energy filter slit width (eV)	20	10	20
Electron exposure per tilt (e^–^/Å^2^)	2	3	3
Total electron dose (e^–^/Å^2^)	~85	~125	~200
Defocus range (μm)	−3	−1.5 to −4.5	−5
Tilt range (degrees)	±60	±60	±60
Tilt step (degrees)	3	3	3
Acquisition scheme	Dose-symmetric	Dose-symmetric	Dose-symmetric
Tilt groups	2	2	2
Pixel size (Å)	1.372	1.56	2.653
Symmetry imposed	N/A	*C*3	*C*3
Initial subtomograms (no.)	N/A	127,874	54,617
Final subtomograms (no.)	N/A	5,161 (EMD-17704) 14,915 (EMD-17708)	6,201
Map resolution (Å)	N/A	7.7 (EMD-17704)	13.4
		8.5 (EMD-17708)	
FSC threshold	N/A	0.143	0.143
Map resolution range (Å)	N/A	7–17 (EMD-17704) 8–16 (EMD-17708)	13.4–297.1

**Extended Data Fig. 7:**
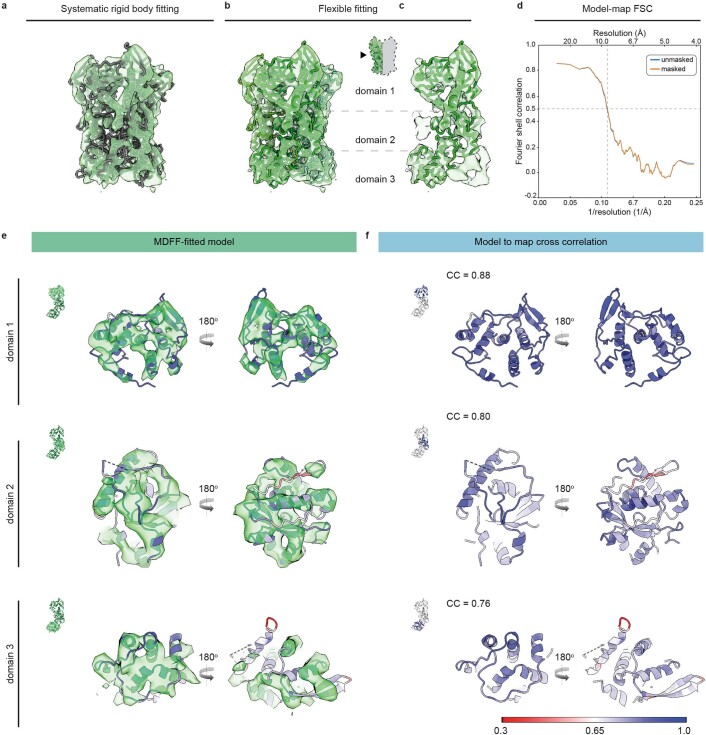
VACV A10 trimer model fitting and validation. (**a**) Model of an AlphaFold2 (AF2)-predicted A10 trimer (dark green) after systematic rigid body fitting into the STA map of a hollow trimer (light green). (**b**) Model of an A10 trimer (dark green) after molecular dynamics flexible fitting (MDFF) into the STA map of a hollow trimer (light green). The model from panel (**a**) was used as an initial template. (**c**) Clipped view of panel (**b**) showing the model fit of one protomer. (**d**) The model-map Fourier shell correlation (FSC) calculated for the model of one protomer shown in panel (**c**). (**e**) Per-domain representation of the A10 protomer fitting into STA map of a hollow trimer. Solvent view is shown on the left, interface view is shown on the right. (**f**) Per-domain representation of the A10 protomer (same as in (**e**)), with residues colored according to the model-to-map cross-correlation (CC) scores calculated in phenix cryoEM validation tool, from the lowest (red) to highest (blue). Solvent view is shown on the left, interface view is shown on the right. The average CC score for each domain is indicated in the upper left corner.

The obtained STA structures indeed correspond to the trimers of A10 protein, as suggested by the result of systematic fitting and subsequent molecular dynamics flexible fitting (MDFF) of the AF2-predicted model of A10 trimer into the STA map of the hollow trimer (Extended Data Fig. 7a–d). This is supported by visual inspection of the secondary structures (Extended Data Fig. 7e), and the model-to-map cross-correlation analysis (Extended Data Fig. 7f). Notably, domain 3, which was predicted with the least confidence by AF2 (Fig. 2a), also showed the lowest cross-correlation with the map (Extended Data Fig. 7f). The MDFF of the A10 trimer model was performed to primarily support the results of systematic fitting. The MDFF introduced changes to the AF2 model, especially at the regions of low resolution. However, we could not conclusively claim that these changes are relevant to the structure, and not due to map imperfections. Therefore, we performed all subsequent model analysis on the basis of the rigid body-fitted AF2-predicted structure.

**Extended Data Fig. 8:**
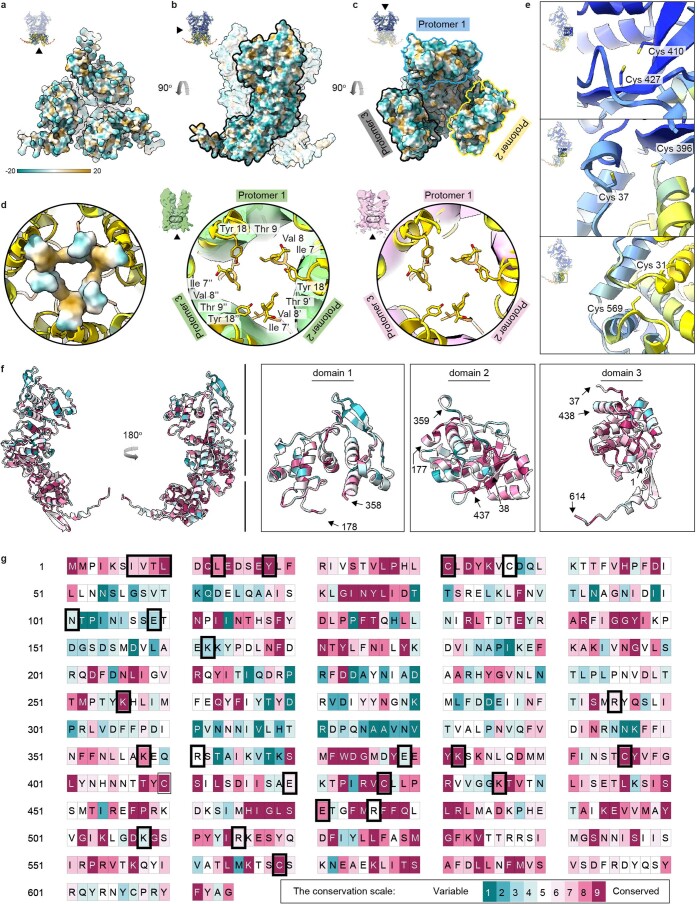
Putative intra- and interprotomer interactions in the A10 trimer. (**a**–**c**) The hydrophobic surface representation of the AF2-predicted model of an A10 trimer showing the bottom view (**a**), side view (**b**), and a top view (**c**). The hydrophobic surface is colored from dark cyan for the most hydrophilic regions to dark gold for the most hydrophobic ones. The values correspond to the molecular lipophilicity potential calculated by the *fauchere* method of the mlp function in ChimeraX based on MLPP program^41^. (**d**) The putative hydrophobic interprotomer interactions within the A10 trimer (left, map coloring is the same as in (**a**–**c**), and slice of the same region showing the AF2 model rigid-body-fitted into densities of the hollow (green) and connected (pink) A10 trimers. (**e**) The cystein residues with the potential of disulfide bond formation mapped on one protomer of the AF2 model of an A10 trimer. Coloring of the model is based on the AF2 confidence score, and is the same as in panel (**d**) and in Fig. 2a. (**f**) Coloring of a protomer based on the residue conservation calculated using ConSurf web server^45^ with default parameters using A10 protomer as a reference. Individual domains of the A10 predicted protomer, corresponding to domain indication in Extended Data Fig. 7 (**b**) and (**c**) are shown as subpanels. (**g**) Sequence representation ConSurf conservation analysis. Residues discussed in the text are highlighted with a black rectangle.

The putative interactions between the protomers within the A10 trimer were addressed by rigid-body fitting of the AF2 model of the trimer into the hollow and connected structures obtained by STA (Fig. 2c,d). This analysis showed that two distinct densities inside the central channel of the respective maps correspond to structured regions of the AF2 model (Fig. 2e,f). First, in the connected trimer, the central channel density at the domain 2 region corresponds to a β-sheet assembly, and predicted putative interactions between Asn101 residues from each protomer (Fig. 2e). Second, in the hollow trimer, the density at the domain 3 region corresponds to predicted hydrophobic interactions between Leu10 and Leu13 of each protomer (Fig. 2f). Notably, the AF2 confidence values were considerably higher for the β-sheet assembly, the main region that defines the connected trimer. Further analysis of a hydrophobic surface (Extended Data Fig. 8a–c) predicted an interprotomer contact in the AF2 model, that was not, however, supported by the density maps, neither for hollow nor for connected trimers (Extended Data Fig. 8d).

The analysis of the AF2-predicted model of the A10 trimer alone revealed a highly positively charged surface in domain 3 oriented towards the virus core wall (Fig. 2g), and a positively charged cavity at the interface between domains 1 and 2 (Fig. 2h). Additionally, it showed regions of potential interprotomer contacts mediated by electrostatic interactions that may provide structural stability of the trimer (Fig. 2i). However, these findings could not be supported by experimental STA densities due to resolution limitations.

### The palisade is an integral part of the incoming core

**Fig. 3:**
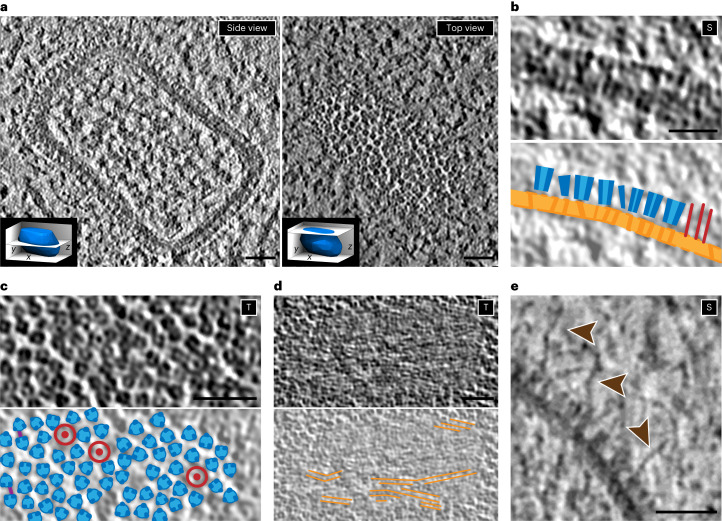
Organization of the in situ VACV core by cryo-ET. **a**–**e**, Digital tomogram slices of in situ VACV cores in the cytoplasm shortly after entry in HeLa cells, showing similar architecture to cores prepared in vitro. **a**, Slices from a central plane (left, side view) and the top (right, top view) of the core. The innermost layer of the core wall observed on in vitro cores is not visible here. Inside the core, filaments without clear organization, potentially the viral double-stranded DNA genome after decondensation, can be observed. Insets indicate the rough positions of the respective tomogram slices with respect to the core. **b**, Details of the side view (S) showing the organization of the palisade layer and the core wall (top) and the corresponding annotation (bottom). The palisade stakes are depicted in blue, the core wall in orange and the side view of a ring-like structure as red lines. **c**, Top view (T) of the palisade layer (top) and its corresponding annotation (bottom). Similar to the in vitro cores, there is no regular arrangement of the stakes (blue). Thin threads (purple lines) connecting the stakes can be observed, as well as the ring-like structures (red circles). **d**, Slice of the top view (T) of the core wall (top) with the corresponding annotation of the stripes that constitute the wall (bottom). **e**, Close-up of a side view (S) at the surface of the palisade, showing strands that potentially represent early transcripts leaving the core (brown arrowheads). Scale bars, 30 nm.

To exclude potential artifacts from the preparation of cores for in vitro study, we compared the structure of the palisade observed on cores in vitro and in situ. To this end, tomograms of cores in the cytoplasm of host cells shortly after virus entry were analyzed. For this, HeLa cells grown on grids were plunge-frozen at 30 minutes postinfection without previous chemical fixation and thinned by focused ion beam (FIB) milling (Methods). Fifteen tilt series were acquired containing cytoplasmic cores. After tomogram reconstruction, the five best-resolved cores were used for averaging of the palisade trimer, as described above for the in vitro cores (Fig. 3).

The overall architecture of the incoming cores observed in situ is very similar to that of the cores analyzed in vitro (Figs. 1 and 3 and Supplementary Video 3). The outer layer represents the prominent palisade (Fig. 3b,c), while the middle layer has a striped appearance, as seen in vitro (Fig. 3b,d). The cytoplasmic cores are devoid of lateral bodies, which are known to dissociate immediately after entry and membrane fusion^6,15^. The electron-dense layer lining the inside of the core wall in the samples analyzed in vitro is not seen in cytoplasmic cores (Fig. 3a). Instead, the central part of the core contains filaments, likely representing decondensed DNA (Fig. 3a), as described previously^14^. A remarkable feature that was not observed in vitro is the presence of strands attached to the palisade, which have been previously reported by Hernandez et al.^11^ and could be early transcripts leaving the core (Fig. 3e). Finally, the ring-like structures located between the palisade stakes found in in vitro cores (Fig. 1c,d) are also observed on the cytoplasmic cores (Fig. 3b,c).

### Comparison of the palisade spike in vitro and in situ

**Fig. 4:**
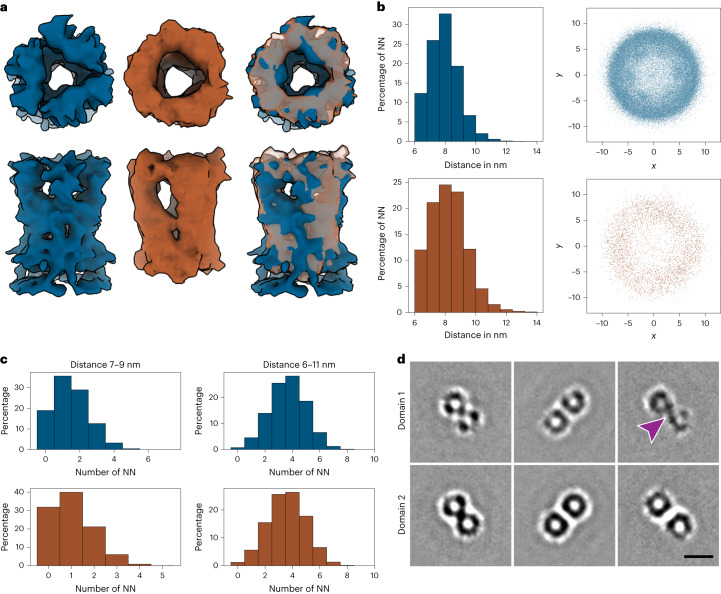
Comparison of in vitro and in situ palisade stakes. **a**, The structures of in vitro (left, blue) and in situ (middle, brown) stakes after localization and alignment (before classification). The right image shows their overlay. While the resolution of the in situ map is lower, the overall shape and size of both structures match very well. **b**, Nearest-neighbor (NN) analysis performed on the stake trimers. Top row (blue), in vitro dataset; bottom row (brown), in situ dataset. Histograms (left) show average distances between the NNs with similar trend for both datasets. The relative position of the NN to the normalized position and orientation of each stake is shown on the right. Positions are shown two-dimensionally for the *x*–*y* dimension. Although minor clustering corresponding to a hexagonal arrangement can be observed in the in vitro dataset, the majority of stakes is not arranged in a regular manner relative to one another. **c**, Analysis of number of NNs within the distance range 7–9 nm (left) and the whole 6–11 nm range (right) for the in vitro (blue) and in situ (brown) datasets. The constrained range corresponds to the average distance between stakes ±1 nm. At this distance, the majority of stakes has only 1–2 neighbors in both datasets. The whole range of distance allows analysis of more NNs and shows that the majority of stakes has only 3–4 NNs. **d**, STA maps of NN pairs at distances 6.5–7.5 nm (left), 7.5–8.5 nm (middle) and 8.5–9.5 nm (right) in the in vitro dataset. The top row shows a cross-section through the top part of domain 1, and the bottom row through domain 2. The stakes do not appear as a trimer, as this shape was averaged out due to lack of strong preferred orientation among the pairs. At the distance range 8.5–9.5 nm (right), a strong density, corresponding to the observed threads from Fig. 1, connects the pair of stakes at the top of domain 1 (purple arrowhead). Scale bar, 10 nm. Source data

A typical challenge with imaging of incoming virus in thin lamellae of infected cells is the limited number of particles available for analysis. Despite this challenge, we were able to resolve the structure of the palisade stakes from the in situ data to 13.4 Å (Extended Data Fig. 6b). This resolution is sufficient to conclude that the size and shape of the palisade from in situ cores are consistent with the in vitro ones when compared at the same resolution (Fig. 4a). Also, on both cores there was no indication of a regular lattice organization for the palisade layer. To further confirm the visual similarity between the in vitro and in situ cores, nearest-neighbor analysis was performed. The average distance between neighboring stakes is 7.9 nm for in vitro and 8.2 nm for in situ datasets, with standard deviations of 0.96 and 1.19, respectively. Analysis of the normalized nearest-neighbor position shows a similar trend (Fig. 4b,c). We also find that there is no visible positional preference of the nearest neighbors relative to another (Fig. 4b), which confirms the irregular arrangements of the palisade lattice observed on cores prepared from purified virus. The number of neighboring stakes within a given distance from the center of a trimer shows that for both datasets only around 5% of the stakes form a hexagonal arrangement (Fig. 4c). Furthermore, the STA maps of pairs of trimers from in vitro cores show that neighboring trimers are not consistently oriented towards each other (Fig. 4d). The average number of stakes per core for the in vitro dataset is 2,690 (with standard deviation of 169) and is based on analysis of 43 cores. Given that the in situ cores are not fully contained within the tomograms acquired on lamellae, we calculated an average stake density on the core surface by dividing the number of observed stakes per core by the surface area of the respective core segmentations, yielding an average of 0.018 stakes per 1 nm^2^ surface for in situ cores and 0.015 stakes per 1 nm^2^ for cores analyzed in vitro.

## Discussion

Among the first steps of the replicative cycle of VACV is fusion of the MV during entry and release of the viral core into the cytoplasm to initiate infection. This core has been studied to quite some extent, both morphologically and biochemically, but the molecular composition of its subunit has remained elusive^6^. Cores can also be prepared from detergent-stripped virus, which facilitated previous studies^10^ and our present analysis. By combining cryo-ET, STA and AF2, we show that the prominent palisade layer is composed of trimers of the gene product of A10. A10 is an abundant VACV core protein that is essential for the formation of the MV^16^; it is synthesized as a 110-kDa precursor that is cleaved to a 65-kDa form during virion maturation^17^. This maturation step is accompanied by formation of the typical oval or brick-shaped core, displaying the palisade on its surface. As described before, the stakes appear as trimers forming hollow tubes^10,11^. In our classification, all 20 classes resulted in an A10 trimer displaying minor differences in protomer orientation. Among these, two global classes can be distinguished, one class presents an open, hollow central part, whereas the other displays densities that connect the protomers across the trimer center. Taken together, this confirms the structural flexibility that can be observed in tomograms where stakes appear to adopt slightly different shapes (Fig. 1). The class with the best-resolved features and highest resolution was used for model fitting with the AF2 prediction used as a starting model. It generally fitted the cryo-ET structure well, although the central channel of the trimer of the native structure, both in vitro and in situ, appeared to be more open. Most importantly, we show that the structure of the stakes on detergent-stripped virions is similar to the one on the incoming core, making us confident that it is physiologically relevant.

We found that the different classes are randomly distributed on the surface of the core without any clustering at specific locations, such as high-curvature regions. Overall, the stakes on both in vitro and in situ cores are randomly organized and display no specific lattice pattern, as shown by nearest-neighbor analysis. This seems to contradict previous data, proposing that the palisade shows both unorganized regions and large patches with a hexagonal arrangement^3^. The difference may originate from the analysis of different maturation stages of the viral replicative cycle: the palisade may be more organized on fully assembled infectious MV^3,11^, whereas it displays no obvious order on intermediates of disassembly, such as membrane-free or cytoplasmic cores.

The core is known to contain at least five abundant proteins (‘Introduction’), four of them being among the most abundant proteins of the MV, namely the gene products of A3, A10, A4 and F17 (ref. ^4^), implying their important structural roles. F17, an 11-kDa protein, is known to be part of the lateral bodies. These may remain attached to cores prepared by gentle detergent stripping, as in this study, but dissociate from the core immediately after entry^6^. AF2 predicts F17 to be generally unordered; as an abundant component of the lateral bodies this fits their amorphous appearance. Our collective in situ and in vitro data allow us to draw the model of the molecular architecture as in Fig. 1a, where the palisade is composed of flexible A10 trimers. Attempts to localize the AF2 predictions of the other major core proteins within the tomograms have been unsuccessful until now. On the basis of the AF2 prediction, the monomer or dimer of A3 could fit, size-wise, into the core wall, which would be consistent with immunolabeling experiments on cytoplasmic cores^6^ and on NP-40–DTT-treated cores, where A3 becomes exposed only after digestion of the cores with proteases^9^. In addition, the patch of strong negative charges on its surface could interact with the base of the A10 trimer, which is positively charged. The latter is consistent with recent cross-linking experiments where A3 was predicted to interact with the C-terminal part of A10 (ref. ^18^). The location of A4, a 39-kDa protein, remains unknown at this stage; previous immunolabeling experiments on cytoplasmic cores would place this protein on the core surface. Indeed, our tomograms occasionally suggested the presence of an additional density on top of the palisade, but its characterization requires further analysis. We also observed the presence of the three pairs of cysteines on each A10 protomer (Extended Data Fig. 8e) that may form disulfide bonds, which in turn could be reduced during viral entry into the host cell. Interestingly, most of these cysteines are highly conserved among other poxviruses (Extended Data Fig. 8f,g).

The identification of the molecular and structural composition of the palisade questions its function, which has been elusive so far. A10 is essential for the formation of the MV and its abundance suggests it shapes the core into its characteristic brick-like shape and links the core to the viral membrane and the lateral bodies. Indeed, we detected direct interactions between stakes and the lateral bodies, as described recently^11^. Accordingly, the palisade could fulfill a scaffolding function similar to the gene product of D13 during membrane assembly, as recently suggested^11^. Being an abundant and flexible protein, we speculate that the stakes have additional functions related to the early replicative cycle, when the palisade is prominently exposed on the core surface. The cytoplasmic core is known to interact with microtubules^19^ and the palisade stakes could mediate this interaction. The rings on the core surface, observed previously^11^ and in the accompanying study, are typically surrounded by A10 trimers. The latter might support and stabilize the rings, contributing to their typical ring-like structure. The subsequent step of early infection is uncoating of the DNA genome and its release into the cytoplasm for replication. EM images documenting this event suggest that genome release occurs by a single and distinct break in the core wall^6,14^. A distinct subset of A10 trimers could facilitate this opening, undergoing rearrangements that locally modify the palisade layer and destabilize the underlying core wall, for release of the viral DNA.

Altogether, by combining cryo-ET, STA and AF2 we have identified the composition of the VACV palisade. The A10 trimer is an abundant and highly flexible protein, with the potential to fulfill multiple functions during VACV assembly and disassembly, which will be the focus of future research.

## Methods

### Mature virions

#### Sample preparation

Mature virus of VACV strain Western Reserve (WR) were purified as previously described^5^. In brief, four plates (24 cm × 24 cm) of HeLa cells at ~80% confluency were infected at a multiplicity of 1 for 3 days. Infected cells were collected by scraping and pelleting and lysed in 10 mM Tris–Cl buffer pH 9 containing 2 mM MgCl_2_ using a dounce homogenizer. After removing cell debris by centrifugation, the virus was pelleted through 36% sucrose (w/v) in 10 mM Tris pH 9, for 30 min at 70,000*g*. Pelleted virus was purified in a continuous 22–32% OptiPrep gradient (w/v) for 30 min at 30,000*g*, and the virus band collected and concentrated by pelleting at 70,000*g*. The virus was quantified by optical density (OD_260_) measurement and the infectious titer was determined by plaque titration. The preparation was sonicated 2 × 30 s in a water bath sonicator and mixed in a 1:1 ratio with 5-nm gold fiducials (Cell Microscopy Core, Utrecht University). This solution was applied to 200-mesh R2/2 copper grids (Quantifoil) and plunge-frozen into liquid ethane/propane cooled down to liquid nitrogen temperature using a Vitrobot Mk4 (Thermo Scientific). For detailed plunge-freezing parameters, see Supplementary Table 1. Vitrified grids were stored in liquid nitrogen until further use.

#### Data collection

At the Multi-User Cryo-EM facility at Centre for Structural Systems Biology (CSSB), the grids were imaged using a Titan Krios G3 (Thermo Scientific) operated at 300 kV and equipped with a BioQuantum-K3 imaging filter (Gatan). Grids were screened and tilt series acquired using SerialEM^20^. The low-dose mode was set up to achieve a dose rate of ~15 e^−^ per pixel per second on the detector directly on the sample with a 70-μm objective aperture and an energy slit width of 20 eV. Tilt series were collected with the dose-symmetric tilt scheme^21^ over a ±60-degree tilt range with a 3-degree increment step. Tilt images were recorded in electron-counting mode at a pixel size of 1.372 Å, a target defocus of −3 µm and an electron dose of 2 e^−^/Å^2^ over ten frames, resulting in a total dose of ~85 e^−^/Å^2^ per tilt series. For an overview of the data acquisition parameters, see Table 1 and Supplementary Table 1.

#### Data processing

Tomograms were processed within eTomo (IMOD v.4.11 package)^22^. Before the reconstruction, the tilt series were aligned using the fiducial-based alignment and dose weighting was applied to remove the damaging frequencies. The tomogram reconstruction was done with 3D-CTF correction and SIRT-like filter equivalent to seven iterations for visualization purposes.

### Cryo-electron tomography of viral cores prepared in vitro

#### Sample preparation

Purified VACV MVs were diluted in 50 mM Tris–Cl pH 8.5 and mixed with an equal volume of 0.2% Nonidet P-40 and 20 mM DTT diluted in Tris–Cl pH 8.5. The mixture was sonicated for 3 × 60 s in a water bath sonicator, then incubated in a ThermoMixer (Eppendorf) for 30 min at 37 °C and 500 r.p.m. and layered on 36% (w/v) sucrose in 50 mM Tris pH 8.5. Cores were pelleted in a table-top centrifuge using a TLS 55 swingout rotor at 100,000*g*, sucrose was removed without disturbing the translucent pellet and remaining sucrose was drained by placing the tube top-down for several minutes on filter paper. These freshly prepared cores were structurally intact and remained associated with lateral bodies, while storing them at −80 °C resulted in a considerable number of broken cores. The fresh pellet was resuspended in 50 mM Tris–Cl pH 8.5 and placed on ice. The preparation was sonicated for 3 × 60 s in a water bath sonicator before mixing with 10-nm gold coupled to protein A (Cell Microscopy Core, Utrecht University) in a 4:1 ratio (cores/gold). This solution was applied to freshly glow-discharged 200-mesh R2/2 copper grids (Quantifoil) and plunge-frozen into liquid ethane using a Vitrobot Mk4 (Thermo Scientific). For the detailed parameters for plunge-freezing, see Supplementary Table 1. Vitrified grids were stored in liquid nitrogen until further use.

#### Data collection

Tilt series data of purified cores were acquired using SerialEM software^20^ on a Titan Krios G4 transmission electron microscope (Thermo Scientific), operated at 300 kV and equipped with an E-CFEG, SelectrisX energy filter and Falcon4 direct electron detector, essentially as described in ref. ^23^. Grids were initially mapped at low magnification in 6 × 6 montages. Intermediate magnification maps of suitable grid squares were collected with a bin 1 pixel size of 19.4 Å, −100-μm defocus, a 70-μm objective aperture and a 20-eV energy slit to identify vitrified VACV cores suspended within foil holes. SerialEM low-dose mode was set up to achieve a dose rate of ~7 e− per pixel per second on the detector, with a 70-μm objective aperture and a 10-eV energy slit inserted. Tilt series data were collected in a dose-symmetric tilt scheme^21^ over a ±60-degree tilt range with 3-degree tilt increments in groups of two tilts. Tilt images were recorded in electron-counting mode at a pixel size of 1.56 Å, a target defocus range of −1.5 to −4.5 µm and an electron dose of 3.0–3.1 e^−^/Å^2^ over ten frames, resulting in a total dose of ~125 e^−^/Å^2^ per tilt series. For an overview of data acquisition parameters, see Table 1 and Supplementary Table 1.

### Cryo-electron tomography of viral cores in situ

#### Sample preparation

To image cores in situ, HeLa cells were seeded onto glow-discharged 200-mesh R1.2/1.3 Au/Au grids (Quantifoil) and grown overnight at 37 °C and 5% CO_2_. Cells were placed in serum-free DMEM high-glucose medium (Gibco) and VACV (WR strain with A3 protein tagged with YFP, kindly provided by M. Way, The Francis Crick Institute, UK) bound for 45 min at room temperature, followed by 30 min of incubation at 37 °C for virus entry. Grids were plunge-frozen in liquid ethane using an EM GP2 device (Leica Microsystems). For detailed plunge-freezing parameters, see Supplementary Table 1. Lamellae were prepared on an Aquilos 2 Cryo-FIB (Thermo Scientific) using Maps and AutoTEM software (Thermo Scientific). Sample preparation, milling and polishing steps were done automatically using AutoTEM with a milling angle target of 8 degrees (2 degrees of tolerance) and final lamella thickness set to 120 nm.

#### Data collection

For tilt series acquisition of in situ cores in lamellae of infected HeLa cells, grids were imaged using a Titan Krios G3 (Thermo Scientific) operated at 300 kV and equipped with a BioQuantum-K3 imaging filter (Gatan). Grids were screened and data acquired using SerialEM. First, grids were mapped at low magnification to localize the lamellae. Mapped grids were pretilted to the angle used during milling (specific for each lamella between 7 and 10 degrees), which was the starting angle for tilt series acquisition. SerialEM low-dose mode was set up to achieve a dose rate of ~15 e^−^ per pixel per second on the detector with a 70-μm objective aperture and a 20-eV energy slit inserted. Tilt series data were collected in a dose-symmetric tilt scheme^21^ over a ±60-degree tilt range around the starting angle with a 3-degree tilt increment. Tilt images were acquired in electron-counting mode at a pixel size of 2.653 Å, a target defocus of −5 µm and an electron dose of 4.5 e^−^/Å^2^ over ten frames, resulting in a total dose of ~200 e^−^/Å^2^ per tilt series. For an overview of data acquisition parameters, see Table 1 and Supplementary Table 1.

### Preprocessing and tomogram reconstruction

The defocus for each tilt series was estimated with CTFFind4 (ref. ^24^). Tilt series were dose weighted using the MATLAB script from ref. ^25^. Suboptimal projections or tilt series were removed from the dataset, resulting in 34 tilt series for the in vitro dataset and 5 tilt series for the in situ dataset. The in vitro tilt series were aligned using fiducial-based alignment, while the in situ tilt series were aligned using patch tracking (both done within eTomo). The tomograms were reconstructed from 8× binned tilt series using WBP with SIRT-like filter option. These tomograms were used only for picking of cores within napari (see below). The unbinned tomograms were reconstructed using novaCTF^26^ with phaseflip correction, astigmatism correction and a 15-nm slab size. These tomograms were binned 2×, 4× and 8× using Fourier3D^27^. These 3D-CTF-corrected tomograms were used for STA. For the complete parameter setup of individual steps, see Supplementary Table 2.

### Subtomogram averaging (in vitro dataset)

#### Creation of initial positions on the core surface

VACV cores were manually segmented by drawing contours around the cores in multiple layers of 8× binned tomograms in napari^28^. The layers were used to create the complete surface of the cores using the triangulation of the respective convex hulls. The resulting surfaces were then used to determine the starting position and orientations for STA (Extended Data Fig. 4k). Two sets of positions were produced for different purposes. The first set was generated for STA with an initial reference. For this set the surface was sampled at a distance of 3.7 nm. The second set of positions was created specifically for resetting normal vector in de novo STA (see below). The surface for this set was sampled with a distance of 1.2 nm.

#### Initial alignment

A total of 400 palisade stakes were manually picked to generate the initial reference (Extended Data Fig. 4b,c) in novaSTA (Supplementary Table 3). Subsequently, STA using all subtomograms was done in novaSTA^29^ on 8× binned tomograms (Supplementary Table 3). After the alignment, overlapping subtomograms were removed from further processing together with the subtomograms located near the carbon edge of the cryo-ET sample support. Visual inspection of the aligned positions showed that most of the stakes were not correctly localized and many subtomograms were aligned into the empty regions among them, as these regions often resembled stakes, shape- and size-wise, at this resolution. A high cross-correlation threshold, which was different for each core, was used to select only subtomograms that were correctly located (Extended Data Fig. 4l) and to obtain an initial STA map from ~8,000 subtomograms, which gave us an estimate of the size and shape of the stakes (Extended Data Fig. 4c).

#### AlphaFold2 prediction

AlphaFold-Monomer v.2.2.0 (ref. ^12^) was used to generate models of four monomers of proteins A3, A4, A10 and L4 (Extended Data Fig. 2). AlphaFold-Multimer v.2.2.0 (ref. ^30^) was used to create multimers of these proteins, as well as their combinations (see Extended Data Table 1 for the full list). The AF2 parameters were set to default, except for the max_recycles parameter, which was set to 12 to ensure convergence of the modeling. The models of the monomers were scored according to the predicted local-distance difference test (pLDDT) score, while the models of protein complexes were scored using a combined interface predicted TM-score and predicted TM-score, as returned by AF2 (Extended Data Table 1).

#### De novo subtomogram averaging

The overall shape of the initial STA map matched the AF2 prediction model of a trimer of A10 (Extended Data Fig. 4d). The AF2 model of the A10 trimer was used as initial reference for STA on the oversampled surfaces. However, to utilize the higher resolution, necessary to distinguish between the stakes and the regions among them, the STA was done on 2× binned tomograms. The AF2 reference was filtered to 15 Å resolution (Extended Data Fig. 4e). The alignment resulted in correctly placed positions (Extended Data Fig. 4m) and a map resembling the structure prediction of the A10 trimer. To avoid template bias, the orientations found during STA were neglected. Instead, each subtomogram was assigned orientation on the basis of its position on the surface: the closest point from the second oversampled set was found and its normal vector was used to assign the cone angle of the subtomogram. The in-plane angles were assigned randomly. An STA average produced with the new orientation resembled a featureless tube (Extended Data Fig. 4f). This was used as a new starting reference for the following STA, together with the particle list of the subtomograms using randomized orientations this time. First, to confirm that the structure has *C*3 symmetry, STA on a subset of particles (9,117) was run using *C*1 symmetry. The resulting map confirmed the trimeric character of the structure (Extended Data Fig. 4g). Next, STA on the full dataset of isolated cores (127,874 subtomograms) was performed applying *C*3 symmetry (Extended Data Fig. 4h). Both maps had different in-plane orientation than the AF2 reference used for particle localization, which confirms that their alignment was not biased by the AF2 reference. The overview of the full parameter setup for both *C*1 and *C*3 is shown in Supplementary Table 3. STA was done using novaSTA and STOPGAP^31^.

#### Classification

Visual inspection of tomograms suggested high flexibility of the stakes based on their nonuniform appearance. The protocol from ref. ^23^ was used to perform classification of the stakes using STOPGAP. In total, 20 de novo initial references were created by choosing random subsets of particles (that is, 10 subsets contained 10,000 particles and 10 subsets contained 5,000 particles). Two independent STA runs of 15 iterations were done using simulated annealing for the first 10 iterations (with temperature factor 10) and random search for the last 5 iterations. All 20 classes resulted in a trimer resembling A10. Visual inspection of the classes revealed that eight classes contained distinct features, separating them from the others (Fig. 4a). These eight classes were used as initial references for four independent STA runs with random search. In total, ten iterations were sufficient for all four runs to stabilize (Fig. 4c). Subsequently, the consistency cleaning was performed: only particles that ended up in the same class for all four runs were kept; the rest were discarded. The remaining particles underwent seven iterations of STA multiclass alignment (that is, without the possibility to change the class during the run). Class 6 (reported resolution 8.5 Å, 14,915 particles) and class 7 (reported resolution 7.7 Å, 5,161 particles) were used for further analysis as the connected trimer and the hollow trimer, respectively. The eight selected classes were mapped back to tomograms using the ArtiaX plugin^32^ in ChimeraX^33^ to visually inspect possible clustering of classes. The model created using ArtiaX showed that there is no defined localization of the subtomogram class given the nonspecific arrangement of its mapped position on the core surface. The Fourier shell correlation (FSC) curves for both class 6 and class 7 are shown in Extended Data Fig. 6a.

### Subtomogram averaging (in situ dataset)

STA of in situ data was performed in the same way as for the in vitro dataset, with the exception of manual picking and initial alignment, which were both skipped. Instead, after segmenting the cores, STA of oversampled positions was performed using the AF2 model of the A10 trimer that was filtered to a resolution of 17 Å. After the localization on the core surface, the randomized orientations were assigned in the same way as for the in vitro data. The new tube-like reference and the particle list with randomized orientation were used for STA (done with STOPGAP). The average was resolved to an overall resolution of 13.4 Å (Fig. 4). The relatively low number of particles (6,201) prevented any attempts of classification. Supplementary Table 3 contains all the parameters applied for the in situ STA. The FSC curves are shown in Extended Data Fig. 6b.

### Systematic fitting of AlphaFold models to cryo-EM maps

We followed the procedure from the work of Zimmerli et al.^34^ to locate the AF2 model in the cryo-EM map of A10. The flexible chain at the N terminus (Met1–Thr9) and at the C terminus (Ser592–Gly614) of the model was removed, since there were no extra densities observed in that region. The AF2 model was filtered to 9 Å and the resulting simulated model map was fitted into cryo-EM maps by global fitting as implemented in UCSF Chimera^35^ using scripts within Assembline^36^. The fitting was performed using 10,000 random initial placements, with at least 30% of the model map covered by the cryo-EM map. To evaluate the fit of each model, we used the cross-correlation about the mean (cam score), which was calculated using UCSF Chimera^35^. We assessed the statistical significance of each fitted model using a *P* value derived from the cam scores. To obtain the *P* values, we first transformed the cross-correlation scores to *z* scores using Fisher’s *z* transform, computed two-sided *P* values on the basis of an empirical null distribution derived from all nonredundant fits and corrected the *P* values for multiple testing using the Benjamini–Hochberg procedure^37^.

### Flexible fitting and model analysis

To improve the fit of the AF2 structure prediction of the A10 trimer, MDFF was performed using both the model obtained by systematic fitting and the STA density map of the hollow trimer as inputs. Flexible fitting was performed on the web server of the Namdinator pipeline tool^38^ in two sequential cycles with the following parameters: map resolution, 12 Å; start temperature, 500 K; final temperature, 298 K; *g*-force scaling factor, 0.3; minimization steps, 5,000; simulation steps, 20,000; Phenix real space refinement cycles, 0; implicit solvent excluded. The obtained model was subjected to Phenix cryo-EM validation tool^39^ to calculate a model-to-map cross-correlation and the model map FSC using default parameters and resolution set to 10 Å. The model-to-map fitting cross-correlation was calculated for the coordinates of one protomer and its corresponding density, as well as for individual domains of the protomer and their respective densities. All densities were segmented in ChimeraX^33^ using the Segger function^40^ based on visual inspection.

Analysis of the A10 interprotomer contacts, specifically hydrophobic interactions between the α-helices Leu10–Leu13 of domain 3 (Fig. 2f) and β-sheets Val90–Leu92, Asn96–Ile99 and Ile104–Ser107 of domain 3 (Fig. 2h), was done using the AF2-predicted model. First, the trimer was fitted as a rigid body into the STA map of a hollow trimer, then the corresponding regions were selected separately, and fitted as two individual rigid bodies into the densities of hollow and connected trimers. The molecular lipophilicity potential was calculated using the default Fauchére method, which specifies how the atomic values propagate through space, with factors based on the distance *d* from the atom. The Fauchére method is integrated into the mlp function in ChimeraX based on the MLPP program^41^. The electrostatic potential was calculated according to Coulomb’s law using the Coulombic surface coloring tool in ChimeraX.

### Reporting summary

Further information on research design is available in the Nature Portfolio Reporting Summary linked to this article.

## Online content

Any methods, additional references, Nature Portfolio reporting summaries, source data, extended data, supplementary information, acknowledgements, peer review information; details of author contributions and competing interests; and statements of data and code availability are available at 10.1038/s41594-024-01218-5.

### Supplementary information

Supplementary Information Supplementary Tables 1–3.

Reporting Summary

Supplementary Video 1 Representative tomogram of purified MV. MVs were purified as described in the Methods. The palisade layer and the core wall underneath the membrane are visible in the otherwise densely packed MV (described in Extended Data Fig. 1). Scale bar, 100 nm.

Supplementary Video 2 Representative tomogram of two cores obtained by detergent stripping of purified virus (Methods). The first core in the sequence of the video shows a core from its elongated side, the second from the lateral side, displaying the lateral bodies that remain attached after the gentle NP-40–DTT treatment used here.

Supplementary Video 3 Representative tomogram of in situ core acquired 30 min postinfection on lamella of infected HeLa cells. The palisade layer and the core wall show similar structures compared to the cores observed in vitro. The filaments underneath the core wall, likely corresponding to the viral dsDNA genome, are rearranged (described in Fig. 3). Scale bar, 100 nm.

### Source data

Source Data Fig. 4 Numerical data to generate plots in Fig. 4.

Source Data Extended Data Fig. 5 Numerical data to generate plots in Extended Data Fig. 5.

## Figures and Tables

**Extended Data Table 1:**
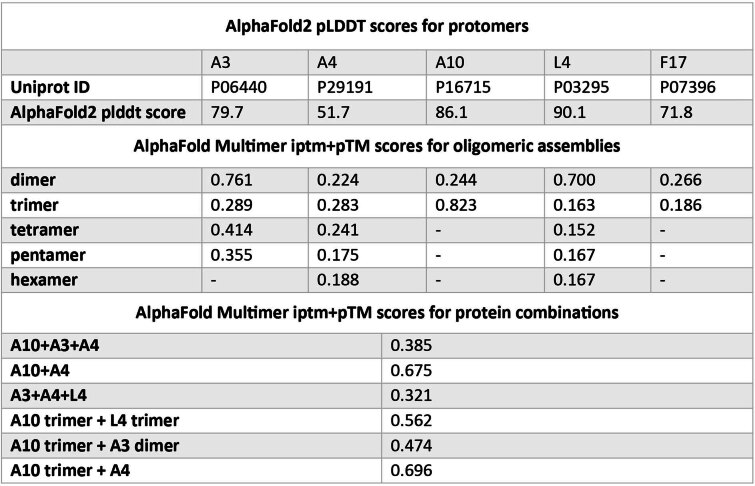
List of VACV proteins, their oligomeric assemblies and combinations analyzed in this study

Each protein is identified by its UniProt ID. The average per-residue confidence score (pLDDT) is given for the highest-ranked model of each of the different proteins. The pLDDT is the confidence measure of AlphaFold2. The score ranges from 100 (indicating perfect confidence) to 0 (indicating no confidence). AlphaFold-Multimer software was used to predict structures of oligomeric assemblies as well as their different combinations with other proteins. The score represents the confidence of the protein–protein interface prediction. It is calculated using the interface pTM (ipTM) score and pTM score. The score ranges from 1.0 (indicating perfect confidence) to 0 (indicating no confidence).

## Data Availability

The electron microscopy density maps of the A10 trimers have been deposited in the Electron Microscopy Data Bank under accession codes: EMD-17704, EMD-17708 and EMD-17753 (hollow and connected conformation from the in vitro dataset, trimer from the in situ data). The raw tilt series of the in vitro dataset, together with representative tomograms from both in vitro and in situ datasets, were deposited at EMPIAR-11674. Source data are provided with this paper.

## Code availability

The Python scripts developed for core picking and contextual analysis are publicly available on github within the cryoCAT^42^ repository (github.com/turonova/cryoCAT) together with Jupyter Notebook showing their usage (github.com/turonova/cryoCAT/tree/main/docs/source/tutorials/surface_oversampling/surface_oversampling.ipynb) and the tutorial (cryocat.readthedocs.io/latest/tutorials/surface_oversampling/surface_oversampling.html).
