# Supplementary material for: The palisade layer of the poxvirus core is composed of flexible A10 trimers
Source: Nat Struct Mol Biol. 2024 Feb 5;31(7):1105–13. doi: 10.1038/s41594-024-01218-5 (PMC11257942; doi:10.1038/s41594-024-01218-5)
Supplement: Supplementary file 1 — Supplementary Tables 1–3. [file 41594_2024_1218_MOESM1_ESM.pdf]

nature structural & molecular biology

Article

https://doi.org/10.1038/s41594-024-01218-5

The palisade layer of the poxvirus core is composed of flexible A10 trimers

In the format provided by the authors and unedited

Supplementary information

Supplementary Table 1. Sample vitrification parameters and additional data acquisition parameters for all datasets. MV, mature virions.

	**Intact MV**	***In vitro* cores**	***In situ* cores**
	**SAMPLE VITRIFICATION**		
**Grid type**	Quantifoil R2/2, 200 mesh Cu/C	Quantifoil R2/2, 200 mesh Cu/C	Quantifoil R1.2/1.3, 200 mesh Au/Au
**Glow discharge**	Pelco easiGlow: 90 sec, 25 mA, residual air, 0.39 mbar	Pelco easiGlow: 90 sec, 25 mA, residual air, 0.39 mbar	Pelco easiGlow: 120 sec, 25 mA, residual air, 0.39m bar
**Vitrification device**	Vitrobot Mk4	Vitrobot Mk4	EM GP2
**Sample volume**	3 µL	3 µL	n/a
**Sample concentration**	$3x10^8$ particles /mL	$1.5x\ 10^{11}$ particles/ mL	n/a
**Nominal blot force**	-1	0	Blot position: 44 and 4.4
**Filter paper**	Whatman 1	Whatman 595	Whatman 1
**Blot time**	3 sec	8 sec	6 sec
**Temperature**	4°C	4°C	38°C
**Relative humidity**	100%	100%	80%
**Wait/drain time**	0 sec	0 sec	0 sec
**Cryogen**	ethane/propane	ethane	ethane/propane
	**DATA ACQUISITION**		
**Microscope**	Titan Krios G3	Titan Krios G4	Titan Krios G3
**Detector**	K3	Falcon4	K3
**Energy filter**	BioQuantum	SelectrisX	BioQuantum
**Objective aperture**	70 µm	70 µm	70 µm
**Dose rate**	~15 e-/pixel/sec	~6 e-/pixel/sec	~15 e-/pixel/sec
**Fractions per tilt**	10	10	10

Supplementary Table 2. Overview of software and parameters used for processing.

Step	Software	Parameters	Values
CTF Estimation	CTFFind4 v.4.1.13 [1]	Pixel size	1.56 Å (*in vitro*)<br>2.65 Å (*in situ*)
		Acceleration voltage	300 keV
		Spherical aberration	2.7 mm
		Amplitude contrast	0.07
		Power spectrum size	512 pixels
		Minimum resolution	30 Å
		Maximum resolution	5 Å
		Minimum defocus	10000 Å
		Maximum defocus	75000 Å
		Defocus step	500 Å
		Astigmatism	100
Dose-exposure correction	Matlab script [2]		
High-peaks removal	eTomo [3] v.4.10.51 (ccderaser)	Peak criterion	10
		Difference criterion	8
		Maximum radius	4.2
		Extra-large difference criterion	19
Cross-correlation alignment	eTomo (tiltxcorr)	Default parameters	
Fiducial model generation (*in vitro*)	eTomo using “Make seed and track” option		
	Seed model (autofidseed)	Default parameters	
	Track beads (beadtrack)	Sobel filter	0.12 – 0.3
		Fill seed model gaps	True
		Local tracking	True
		Local area size	1000
Fiducial model generation (*in situ*)	eTomo using “Use patch tracking to make fiducial model” option		
	Patch Tracking	Size of patches	500,500
		Fractional overlap of patches	0.8,0.8
		Iterations to increase subpixel accuracy	4
	Filters	Low frequency rolloff sigma	0.01
		High frequency cutoff radius	0.08
		High frequency rolloff sigma	0.05
Alignment transformation computation	eTomo (tiltalign)	Do not sort fiducial into 2 surfaces	True
		Rotation solution type	One rotation
		Magnification solution type	Fixed at 1.0
		Tilt angle solution type	Fixed
		Distortion solution type	Disabled
		Beam tilt	No
Preliminary 8x binned reconstruction	eTomo (tilt)	Logarithm of densities	No
		Radial filtering cutoff	No
		Radial filtering falloff	No
		SIRT-like filter	15 iterations
Reconstruction	novaCTF [4]	Correction type	Phaseflip
		Astigmatism correction	True

		Slab size	15 nm
Binning	Fourier3D [5]		
Core picking	Napari [6]		
Subtomogram Averaging	novaSTA [7]<br><br>STOPGAP [8]	See Table S3	
FSC	STOPGAP		
Systematic Fitting	UCSF Chimera [9] together with scripts within Assembline [10]	Resolution<br>Calpha atoms only<br>Backbone atoms only<br>Move PDB to center<br>Placement number	9 Å<br>False<br>False<br>True<br>10000
Flexible Fitting	Namdinator [11]	See the methods section	

Supplementary Table 3. Overview of software and parameters used for subtomogram averaging (STA).

**STA parameters (*in vitro*)**	**Manual picking**	**Oversampled positions with manually picked ref**	**Oversampled positions with AF2 ref**	***De novo* STA symmetry estimation**	***De novo* STA**
Voxel size	12.48 Å	12.48 Å	3.12 Å	3.12 Å	3.12 Å
Box size (voxels)	48	48	96	96	96
Iterations	10	5	1	5	7
Cone angle increment (degrees)	4	4	4	6 3 3 2 2	6 3 3 2 2 2 2
Cone angle iterations	12	12	12	4 3 3 3 1	4 3 3 3 1 1 2
In-plane angle increment (degrees)	6	6	12	12 12 6 3 30	12 12 6 3 30 10 5
In-plane angle iterations	4	4	5	15 15 15 15 6	5 5 5 5 6 6 6
Low-pass filter (voxels)	20	20	20	12 24 24 30 32	12 24 24 30 32 34 34
High-pass filter (voxels)	1	1	1	1	1 1 1 1 1 1 1
Symmetry applied	1	1	3	1	3
Particle number	400	194978	748260	9117	127874
Particle number after CC cleaning	x	7551	127874	n/a	n/a
Software	novaSTA	novaSTA	STOPGAP	STOPGAP	STOPGAP

**STA parameters (*in situ*)**	**Oversampled positions with AF2 initial reference**	***De novo* STA**
Voxel size	5.3 Å	5.3 Å
Box size (pixels)	96	56
Iterations	1	7
Cone angle increment (degrees)	4	6 3 3 2 2 2 2
Cone angle iterations	12	4 3 3 3 1 1 2
In-plane angle increment (degrees)	12	12 12 6 3 30 10 5
In-plane angle iterations	5	5 5 5 5 6 6 6
Low-pass filter (pixels)	30	12 14 14 18 18 22 22
High-pass filter (pixels)	1	1 1 1 1 1 1 1 1
Symmetry applied	3	3
Particle number	54617	6201
Particle number after CC cleaning	6201	n/a
Software	novaSTA	STOPGAP

SI References:

[1] A. Rohou and N. Grigorieff, ‘CTFFIND4: Fast and accurate defocus estimation from electron micrographs’, *J. Struct. Biol.*, vol. 192, no. 2, pp. 216–221, Nov. 2015, doi: 10.1016/j.jsb.2015.08.008.
[2] W. Wan *et al.*, ‘Structure and assembly of the Ebola virus nucleocapsid’, *Nature*, vol. 551, no. 7680, pp. 394–397, Nov. 2017, doi: 10.1038/nature24490.
[3] J. R. Kremer, D. N. Mastronarde, and J. R. McIntosh, ‘Computer Visualization of Three-Dimensional Image Data Using IMOD’, *J. Struct. Biol.*, vol. 116, no. 1, pp. 71–76, Jan. 1996, doi: 10.1006/jsbi.1996.0013.
[4] B. Turoňová, F. K. M. Schur, W. Wan, and J. A. G. Briggs, ‘Efficient 3D-CTF correction for cryo-electron tomography using NovaCTF improves subtomogram averaging resolution to 3.4Å’, *J. Struct. Biol.*, vol. 199, no. 3, pp. 187–195, Sep. 2017, doi: 10.1016/j.jsb.2017.07.007.
[5] turonova, ‘turonova/Fourier3D: Fourier3D’. Zenodo, Aug. 05, 2020. doi: 10.5281/zenodo.3973621.
[6] N. Sofroniew *et al.*, ‘napari: a multi-dimensional image viewer for Python’. Zenodo, Nov. 03, 2022. doi: 10.5281/zenodo.7276432.
[7] turonova, ‘turonova/novaSTA: novaSTA’. Zenodo, Aug. 05, 2020. doi: 10.5281/zenodo.3973623.
[8] W. Wan, S. Khavnekar, J. Wagner, P. Erdmann, and W. Baumeister, ‘STOPGAP: A Software Package for Subtomogram Averaging and Refinement’, *Microsc. Microanal.*, vol. 26, no. S2, pp. 2516–2516, Aug. 2020, doi: 10.1017/S143192762002187X.
[9] E. F. Pettersen *et al.*, ‘UCSF Chimera--a visualization system for exploratory research and analysis’, *J Comput Chem*, vol. 25, no. 13, pp. 1605–1612, Oct. 2004, doi: 10.1002/jcc.20084.
[10] V. Rantos, K. Karius, and J. Kosinski, ‘Integrative structural modeling of macromolecular complexes using Assembline’, *Nat. Protoc.*, vol. 17, no. 1, pp. 152–176, Apr. 2022, doi: 10.1038/s41596-021-00640-z.
[11] R. T. Kidmose, J. Juhl, P. Nissen, T. Boesen, J. L. Karlsen, and B. P. Pedersen, ‘Namdinator – automatic molecular dynamics flexible fitting of structural models into cryo-EM and crystallography experimental maps’, *IUCrJ*, vol. 6, no. 4, pp. 526–531, Jul. 2019, doi: 10.1107/S2052252519007619.
